# Hydrogels Based on Poly(Ether-Ester)s as Highly Controlled 5-Fluorouracil Delivery Systems—Synthesis and Characterization

**DOI:** 10.3390/ma14010098

**Published:** 2020-12-28

**Authors:** Adam Kasiński, Monika Zielińska-Pisklak, Ewa Oledzka, Grzegorz Nałęcz-Jawecki, Agata Drobniewska, Marcin Sobczak

**Affiliations:** 1 Department of Biomaterials Chemistry, Chair of Analytical Chemistry and Biomaterials, Faculty of Pharmacy, Medical University of Warsaw, 1 Banacha St., 02-097 Warsaw, Poland; adam.kasinski@wum.edu.pl (A.K.); monikapisklak@o2.pl (M.Z.-P.); ewa.oledzka@wum.edu.pl (E.O.); 2 Department of Environmental Health Sciences, Faculty of Pharmacy, Medical University of Warsaw, 1 Banacha St., 02-097 Warsaw, Poland; grzegorz.nalecz-jawecki@wum.edu.pl (G.N.-J.); agata.drobniewska@wum.edu.pl (A.D.)

**Keywords:** biomedical hydrogels, drug delivery systems, 5-fluorouracil, polymeric drug carriers, ε‑caprolactone, *rac*‑lactide

## Abstract

A novel and promising hydrogel drug delivery system (DDS) capable of releasing 5‑fluorouracil (5-FU) in a prolonged and controlled manner was obtained using ε‑caprolactone‑poly(ethylene glycol) (CL-PEG) or *rac*‑lactide-poly(ethylene glycol) *(rac‑*LA-PEG) copolymers. Copolymers were synthesized *via* the ring-opening polymerization (ROP) process of cyclic monomers, ε‑caprolactone (CL) or *rac*-lactide (*rac-*LA), in the presence of zirconium(IV) octoate (Zr(Oct)_4_) and poly(ethylene glycol) 200 (PEG 200) as catalyst and initiator, respectively. Obtained triblock copolymers were characterized by nuclear magnetic resonance (NMR) and gel permeation chromatography (GPC) techniques; the structure and tacticity of the macromolecules were determined. The relationship between the copolymer structure and the reaction conditions was evaluated. The optimal conditions were specified as 140 °C and 24 h. In the next step, CL-PEG and *rac-*LA-PEG copolymers were chemically crosslinked using hexamethylene diisocyanate (HDI). Selected hydrogels were subjected to *in vitro* antitumor drug release studies, and the release data were analyzed using zero-order, first-order, and Korsmeyer-Peppas mathematical models. Controlled and prolonged (up to 432 h) 5-FU release profiles were observed for all examined hydrogels with first-order or zero-order kinetics. The drug release mechanism was generally denoted as non-Fickian transport.

## 1. Introduction

Tumors are the diseases that have most affected human health in recent years. According to WHO statistics, cancer is the second leading cause of death worldwide and is responsible for an estimated 9.6 million deaths in 2018. Globally, approximately one in six deaths is due to cancer. Every year, the number of people who died from tumors has risen significantly. It is possibly due to an aging population and changes in the living environment and daily habits. The most common cancers are lung, breast, colorectal, prostate, skin cancer (non-melanoma), and stomach. In turn, the most common causes of cancer death are: lung, colorectal, stomach, liver, and breast cancers [1].

5-FU (5-fluoropyrimidine-2,4-[1H,3H]-dione) is a cytotoxic drug from the group of antimetabolites [2,3,4]. 5-FU has a broad spectrum of anti-cancer activity. It is used both in monotherapy and in combined therapy of malignant neoplasms, primarily colon, rectal, breast, stomach, pancreatic or skin cancer, and in Bowen’s disease or other precancerous conditions [5,6].

Although intravenous infusion is the most widely used form of 5-FU, numerous attempts are being made to develop alternative dosage forms of this drug. Among them, the most common are polymeric innovative drug delivery systems, such as implants [7,8], microspheres [9,10,11], nanoparticles [12,13,14], macromolecular prodrugs [15,16], or hydrogels [17,18,19,20]. The use of such solutions is mainly aimed at enabling targeted therapy, limiting the biodistribution of the active drug to the target tissue only and reducing its systemic concentration. This strategy increases the effectiveness of treatment and reduces side effects. The desirable characteristics of drug delivery systems (DDSs) are sustained and controlled release profiles, which increase the efficacy and safety of therapy and also enhance patient comfort and compliance [20]. In addition, special efforts are being made to achieve release kinetics as close as possible to zero-order kinetics.

Hydrogels are one of the kinds of prospective anti-cancer drug carriers. Owing to the unique properties of hydrogels, researchers have made significant progress in tumor model reconstruction, tumor diagnosis, and therapy. Notably, hydrogel-based systems can be adjusted to respond to cancer-specific hallmarks and/or external stimuli (e.g., enzyme, antigen, heat, pH, light, and ultrasound). In addition, a hydrogel carrier can cause less serious side effects than systemic chemotherapy and may result in a sustained delivery of the drug at tumor sites. Furthermore, hydrogels have excellent biocompatibility and biodegradability and low toxicity [21].

One of the key stages of hydrogel formation is crosslinking, which can be physical or due to the chemical reactions, creating interactions between the gel-forming macromolecules [21,22,23,24]. Among the polymers used in the hydrogel technology, both natural and synthetic materials are used. Natural polymers, although commonly used as gel-forming materials, are difficult to process and have worse mechanical properties than synthetic ones [25]. However, special attention should be given to the biodegradability and biocompatibility of these systems when used as biomedical devices.

Nowadays, very extensive research is being conducted on the use of hydrogel matrices as DDSs, including antitumor drugs. Hydrogels are capable of releasing hydrophilic and hydrophobic drugs; also, gel-forming macromolecules serve as solubilizers for insoluble substances. There are few major strategies for obtaining hydrogel DDSs. It is possible to simply dissolve the drug into a polymer solution to form a drug-loaded hydrogel; the drug can be loaded into self-forming micelles of macromolecules [26]. The other strategy is to create chemically bonded macromolecular prodrugs capable of forming hydrogels in aqueous media [27]. On the other hand, there are some developments when the hydrophobic active substances are loaded into special carriers (e.g., nanoparticles) and subsequently incorporated into a gel-forming matrix [25].

So far, numerous 5-FU hydrogel delivery systems have been established. Among them, there are developments such as hydrogels [28,29], injectable hydrogels [26], nanogels [18], and smart hydrogels sensitive to various stimuli [27,30], which seem to be promising carriers of antitumor drugs. However, due to the hydrophilic properties of 5-FU, one of the major problems associated with 5‑FU‑releasing hydrogels are relatively rapid drug release with not zero-order kinetics. In addition, some of these systems are not biodegradable, which is a serious drawback in biomedical applications [31,32]. In recent years, biodegradable and biocompatible polymers of cyclic esters [33,34], such as ε‑caprolactone (CL), *rac*-lactide (*rac*-LA), L,L-lactide (LL-LA), or glycolide (GA), are extensively studied in various biomedical applications, including hydrogel drug delivery systems releasing antitumor drugs. Hydrogels as 5-FU DDSs are defined in the literature, but reports on biodegradable, especially triblock CL-PEG (glycol polyethylene) and LA-PEG copolymers such as 5-FU hydrogel DDSs are limited. To date, reports on these materials are mainly based on physically crosslinked matrices, which allow the rapid drug release and following first-order kinetics; burst release was also observed [35,36]. The synthesis of CL-PEG and LA-PEG copolymers is usually carried out *via* ROP reaction, using a stannous octoate (Sn(Oct)_2_) as catalyst. However, zirconium-based catalysts have a reduced toxicity when compared to Sn(Oct)_2_; they are described as inert and eliminated with urine and bile [37].

In this study, we report the synthesis of novel hydrogels as potential carriers of a hydrophilic antitumor agent; 5-FU has been used as a model drug. The goal of the research was to develop an alternative antitumor DDS capable of releasing 5-FU in a prolonged and controlled manner with reduced burst release. Hydrogels have been obtained from copolymers of CL (or *rac-*LA) and PEG, which have been chemical crosslinked with 1,6‑diisocyanatohexane (HDI). The copolymers have been synthesized *via* the ring-opening polymerization (ROP) process in the presence of stannous zirconium (Zr(Oct)_4_) as catalyst. The resulting crosslinking polymers were tested to release 5-FU from them. The rate of drug release from the synthesized DDSs has been analyzed *in vitro*. It is suspected that the obtained hydrogels will show prolonged and controlled drug release profiles, and the release kinetics will be close to zero order.

## 2. Materials and Methods

### 2.1. Materials

ε‑Caprolactone (2‑Oxepanone, CL, 99%, Aldrich, Poznan, Poland), 3,6‑dimethyl‑1,4‑dioxane‑2,5‑dione (*rac*‑lactide, *rac*‑LA, 99%, Sigma-Aldrich, Poznan, Poland), zirconium(IV) 2‑ethylhexanoate (zirconium octoate, Zr(Oct)_4_, Sigma‑Aldrich, Poznan, Poland), toluene (99.8%, POCh, Gliwice, Poland), hydrochloric acid (HCl, ChemPur, Piekary Slaskie, Poland), stannous 2‑ethylhexanoate (stannous octoate, Sn(Oct)_2_, 95 %, Aldrich, Poznan, Poland), dichloromethane (DCM, CH_2_CL_2_, ≥99.8%, POCh, Gliwice, Poland), 1,6‑diisocyanatohexane (hexamethylene diisocyanate, HDI, 98%, Aldrich, Poznan, Poland), poly(ethylene glycol) 200 (PEG 200, pure, Fluka, Warsaw, Poland), poly(ethylene glycol) 600 (PEG 600, pure, Fluka, Warsaw, Poland), 5‑fluorouracil (5-FU, 99%, TCI, Zwijndrecht, Belgium), phosphate-buffered saline (PBS, pH 7.00 ± 0.05, ChemPur, Piekary Slaskie, Poland), and trifluoroacetic acid (TFA, 99%, Sigma-Aldrich, Poznan, Poland) were used as received.

### 2.2. NMR Data

The ^1^H NMR spectrum of CL-PEG: 1.38 ppm (‑O‑CH_2_-CH_2_-C**H_2_-**CH_2_-CH_2_**-**), 1.65 ppm (-O-CH_2_-C**H_2_**-CH_2_-C**H_2_**-CH_2_-), 2.31 ppm (-O(O)C-C**H_2_**-CH_2_-CH_2_-CH_2_-CH_2_-), 3.65 ppm (-O-C**H_2_**-C**H_2_**-) from PEG mers and (‑CH_2_‑CH_2_-CH_2_-CH_2_-C**H_2_**-OH end groups), 4.06 ppm (-O-C**H_2_**-CH_2_-CH_2_-CH_2_-CH_2_-) and 4.23 ppm (-O-CH_2_-C**H_2_**-O(O)C-CH_2_-CH_2_-CH_2_-CH_2_-CH_2_-). The ^13^C NMR spectrum of CL-PEG: 24.7 ppm (-O(O)C-CH_2_-**C**H_2_-CH_2_-CH_2_-CH_2_-), 25.6 ppm (-O(O)C-CH_2_-CH_2_-**C**H_2_-CH_2_-CH_2_‑), 28.4 ppm (-O-CH_2_-**C**H_2_-CH_2_-CH_2_-CH_2_-), 34.2 ppm (-O(O)C-**C**H_2_-CH_2_-CH_2_-CH_2_-CH_2_-), 64.2 ppm (-O-**C**H_2_-CH_2_-CH_2_-CH_2_-CH_2_-), 70.6 ppm (-O-**C**H_2_-**C**H_2_-O-), and 173.6 ppm (-O(O)**C**-CH_2_-CH_2_-CH_2_-CH_2_-CH_2_-).

The ^1^H NMR spectrum of *rac*‑lactide-poly(ethylene glycol) (*rac*-LA-PEG): 1.57 ppm (-O(O)C-CH(C**H_3_**)-), 3.63 ppm (‑O‑C**H_2_**‑C**H_2_**‑O‑), 4.29 ppm (-O(O)C-C**H**(CH_3_)-OH end group), and 5.17 ppm (‑O(O)C-C**H**(CH_3_)-). ^13^C NMR spectrum of *rac*-LA-PEG: 16.7 ppm (-O(O)C-CH(**C**H_3_)-), 69.0 ppm (-O(O)C-**C**H(CH_3_)-), 70.5 ppm (‑O‑**C**H_2_‑**C**H_2_‑O‑) and 169.3 ppm (-O(O)**C**-CH(CH_3_)-). 13C NMR signals used for microstructure analysis: 69.14 ppm (*sis, iii, iis, sii* tetrads), 69.31 ppm (*isi, iss* tetrads), 69.45 ppm (*sss* tetrads), 69.55 ppm (*ssi* tetrads).

### 2.3. Synthesis of Copolymers CL, LA and PEG

First, 1.0 mL CL or 1.0 g *rac-*LA were placed into 10 mL glass ampoules under argon atmosphere, and 1.0 mL of toluene was added. In the next step, catalyst Zr(Oct)_4_ and initiator (PEG 200) were added. Monomer/initiator and initiator/catalyst ratios were constant and equal to 100:1 and 2:1, respectively. The tubes were sealed and thermostated in an oil bath by 24–48 h, and the temperatures was in the range of 60–80 °C. In the other process, polymerization was carried out in melt, and the temperature range was 100–160 °C.

When the reaction was completed, the cooled product was dissolved in DCM and extracted with 5% hydrochloric acid (HCl) and distilled water. Subsequently, the organic phase was evaporated, and the final product was dried in a vacuum to isolate the powder or oil polymer.

The process scale was extended, and the reaction was conducted using 5.0 g or 5.0 mL of monomer, when the synthesis parameters (24 h, 140 °C) were optimized.

In addition, CL‑PEG and *rac-*LA‑PEG copolymers were synthesized within 1–4 h to evaluate polymerization kinetics at the beginning of the reaction.

### 2.4. Preparation of Hydrogels

Hydrogels have been prepared by chemical crosslinking with HDI. The reaction was carried out in an open system. A beaker was placed in a 70 °C oil bath equipped with a thermometer, filled with a calculated amount of PEG 600, and left for 20 min to equalize the temperature. Subsequently, 0.5 g of copolymer was added, and a catalytic amount of Sn(Oct)_2_ was added dropwise after 20 min. After 5 min, the solution was combined with HDI and left in 70 °C for 24 h.

### 2.5. In Vitro Studies of 5-FU Release from Hydrogels

#### 2.5.1. 5‑FU Loading

5-FU was loaded to the hydrogel matrices by physical mixing due to the following procedure. Around 5.0 % (m/m) of 5-FU was added to the crosslinked hydrogel sample and mixed with DCM to suspend the 5-FU and dissolve the matrix. The mixture was dried in vacuum at room temperature to obtain a drug-loaded hydrogel film.

#### 2.5.2. 5-FU Release Studies

*In vitro* 5-FU release experiments were performed in 37 °C with PBS exchange. Vials containing hydrogel films were filled with 4.0 mL of PBS buffer (pH 7.00 ± 0.05), sealed, and left in 37 °C for 2 h. Then, the solution was removed for further testing and replaced with fresh PBS. Subsequent samples were obtained at selected intervals. Release studies have been conducted for 600 h.

### 2.6. Measurements

#### 2.6.1. Structural Analysis

The synthesized copolymers structure, PEG content, and microstructure (*rac-*LA-PEG) were characterized using ^1^H and ^13^C NMR techniques (Varian 300 MHz, Palo Alto, CA, USA). The spectra were registered in CDCl_3_ and DMSO-d_6_. The ^1^H spectra were recorded under following conditions: 300 MHz, 1 s repetition time, 2 s acquisition time, 32, 64 or 128 scans. ^13^C: 75.4 MHz, 5 s repetition time, 1.4 s acquisition time, 20,000 scans per each spectrum. The percentage molar amount of PEG in a copolymer chain was estimated using the following formulas:

For CL-PEG:

(1) $$x=\frac{\frac{I_{PEG}}{2}}{I_{\varepsilon }+\frac{I_{PEG}}{2}}\;\cdot 100\%$$

For *rac*‑LA‑PEG:

(2) $$x=\frac{\frac{I_{PEG}}{4}}{I_{\alpha }+\frac{I_{PEG}}{4}}\;\cdot 100\%$$

Transesterification coefficient (*T*) was determined with the ^13^C NMR spectra of *rac‑*LA‑PEG copolymers.

The average molecular weight and molecular weight distribution were determined on a LabAlliance gel permeation chromatograph equipped with Jordi Gel DVB mixed bed (250 mm × 10 mm) column and refractive detector, using chloroform as solvent. The flow rate was 1 mL/min. The average molecular weights were calibrated with polystyrene standards.

Acute toxicity of the obtained copolymers was determined with Spirotox and Microtox tests, using luminometer Microtox M500 (ModernWater, New Castle, PA, USA).

Crosslinked hydrogels were tested for the water absorption abilities. Approximately 0.5 g of polymer was placed in a 10 mL beaker and dissolved in DCM. Subsequently, the solvent was evaporated and the mass of the polymer film obtained was calculated. Afterwards, 4.0 mL of water was applied and left at room temperature for 7 days. In the next step, the unabsorbed amount of water was decanted, the residue mass was determined, and the percentage of water absorption ability was calculated.

#### 2.6.2. HPLC Analysis

Preliminary 5‑FU determination conditions were developed using a Dionex system consisting of a pump type 7580, Jetstream II Plus (WO Industrial Electronics, Vienna, Austria) thermostat, DAD-detector UVD 340S. A pre-column Phenomenex C‑18 4 mm × 3 mm and column Phenomenex Luna C‑18 25 cm × 4.6 mm (particle size 5 µm) were used. The analytical wavelength was 266 nm according to the literature [38]. The mobile phase was composed of solvent A (H_2_O + 0.1% TFA) and solvent B (acetonitrile (ACN) + 0.1% TFA), and the gradient conditions were described in Table S1 (Supplementary Materials).

Column temperature was 35 °C, the injection volume was 20 µL, and the flow rate was 1.0 mL/min. The described method was moved to a Hitachi Chromaster system consisting of a pump type 5160, thermostat 5310, DAD-detector UVD 5430, and autosampler model 5260. The method was validated, and the concentrations of 5‑FU were determined.

### 2.7. Mathematical Models for 5‑FU Release Studies

The release data points were subjected to zero-order and first-order kinetics and Higuchi and Korsmeyer–Peppas models. Calculations were made on the basis of formulas:

Zero-order model:

(3) F=kt

First-order model:

(4) $$\log F=\log F_{0}-\frac{kt}{2.303}$$

Korsmeyer–Peppas model:

(5) $$F=kt^{n}\;(F<0.6)$$

where *F* is the fraction of 5-FU released up to time (*t*), *F*_0_ is the initial concentration of 5-FU, *k* is the constant of the mathematical models, and *n* is the exponent of the Korsmeyer–Peppas model [39].

## 3. Results and Discussion

**Scheme 1:**
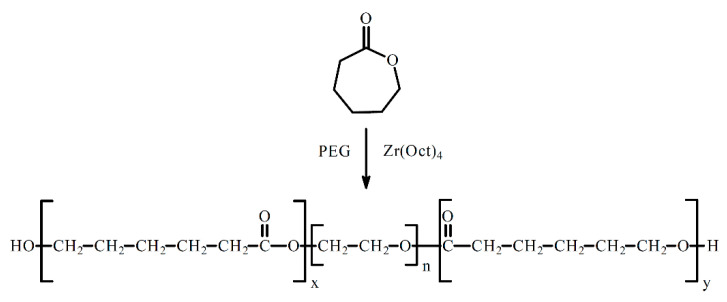
Ring-opening polymerization of ε‑caprolactone (CL) using zirconium(IV) octoate (Zr(Oct)_4_) and poly(ethylene glycol) 200 (PEG 200).

The goal of this research was to obtain polyester-based hydrogels as 5-FU carriers. The hydrogels were synthesized by two steps. Firstly, ROP of CL (or *rac-*LA) and PEG was carried out with Zr(Oct)_4_ as a catalyst (Scheme 1).

The ROP process was carried out in toluene or *via* the bulk method (Table 1 and Table 2).

The *M_n_* of the synthesized copolymers of CL-PEG was in the range of 2800–13,600 g/mol, depending on the reaction parameters and method of synthesis; the dispersity index (*Đ*) varied from 1.49–2.42. For the reactions carried out in toluene, it has been found that the increase in reaction time and temperature increases both *M_n_* and *Đ*. The reproducibility of the process was satisfactory, the differences in these parameters for two samples synthesized under the same conditions being 200 g/mol and 0.04, respectively. Similar relationships were observed for the synthesis carried out in bulk, but the *M_n_* and *Đ* values were higher than those in toluene. We assume that these parameters do not depend on the synthesis method but are related to the higher temperatures used for the reactions in bulk.

In turn, the *M_n_* of the copolymers of *rac*-LA-PEG was in the range of 2300–11,700 g/mol with *Đ* ranged from 1.62 to 2.64. It is presumed that high *Đ* values are associated with high temperature and a long reaction time. It was noted that the increase in these parameters increases *Đ*. It is suspected that at high temperatures, the propagation rate is much higher than the initiation rate of the polymerization process, and the prolonged reaction time exacerbates this effect. Elevated *Đ* values were noted for another zirconium-based catalyst when the reaction was carried out under similar conditions [40]. For the reaction carried out in optimized conditions (140 °C, 24 h), the *Đ* values were acceptable. It has been found that *M_n_* of these copolymers is slightly lower when compared to CL-PEG copolymers and the *Đ* is higher, although the overall results are similar. The major observed difference between two methods of synthesis is correlated with the reaction yield. The process conducted in toluene showed very low efficiency, so bulk-method synthesis was considered as a preferred method, providing the optimum product with desired yield.

The structure of the CL-PEG and *rac*-LA-PEG copolymers was analyzed by ^1^H or ^13^C NMR. The characteristic signals observed on the spectra confirmed the structure of obtained copolymers. ^1^H and ^13^C NMR spectra of CL‑PEG and *rac*-LA-PEG copolymers are shown in Figures S1–S4 (Supplementary Materials).

**Figure 1:**
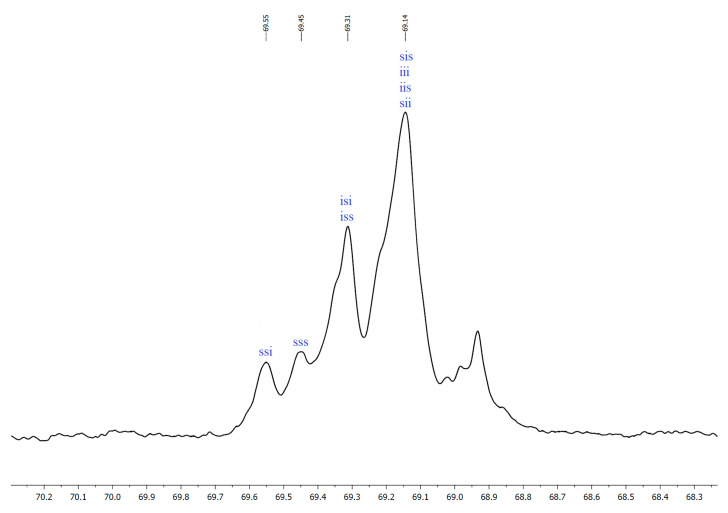
The ^13^C NMR spectrum of *rac*‑lactide-poly(ethylene glycol) (*rac*-LA-PEG) microstructure analysis (in CDCl_3_).

The microstructure of *rac*-LA-PEG copolymers was evaluated by ^13^C NMR spectra with the tetrads distribution (for the methine carbon) (Figure 1). When the intermolecular transesterification process does not occur during ROP, the resonance lines, due to *iss*, *sss*, and *ssi* tetrads, have not been observed in the methine region. The values of transesterification coefficient (*T*) were determined from the proportion of *iss* tetrad using Bernoullian statistics. The experimental *isi* relative weight can essentially vary from 0.125 (random linkage of lactyl units) to 0.25 (Bernoullian addition of pairs). It is known that the *T* values vary from 0 to 1, and in a stereoselective process, the upper limit related to the *isi* tetrad relative weights is higher [41].

The *T* values observed for *rac*-LA-PEG copolymers synthesized in toluene ranged from 0.72 to approximately 1 (Table 2). The increase in temperature and time of the process has been observed to minimize the transesterification coefficient. It is assumed that in the conditions mentioned, the rate of polymerization is higher than the rate of transesterification reactions, and hence, the observed *T* values are lower. When polymerization has been done under bulk conditions, the dependences are slightly different. The *T* value tends to be independent of time and temperature (up to 140 °C) and ranges from 0.32 to 0.72. As the reaction temperature reaches 160 °C, the effect becomes significant and the *T* value increases. What is significant is that the transesterification coefficients for bulk reactions are evidently lower compared to the toluene process; therefore, this method of synthesis was considered to be preferred. However, all synthesized *rac*-LA-PEG copolymers have significant *T* values; thus, their microstructure must be defined as atactic. Since intense transesterification takes place, no conclusions can be made on the stereoselectivity of Zr(Oct)_4_ as an ROP catalyst. However, it can be concluded that the catalyst system (in certain monomer/co-initiator and co-initiator/catalyst molar ratios) promotes the occurrence of a transesterification side reactions.

In the next step of our study, the CL-PEG and *rac*-LA-PEG copolymers were obtained in a larger scale (Table 3).

Optimal process conditions were defined as 140 °C and 24 h, and bulk-method synthesis was considered to be preferred. Under these conditions, the co-initiator content in the macromolecules was the lowest, and the reaction continued with the optimum yield. In addition, the *rac-*LA-PEG copolymers were distinguished by a low *T* factor.

Cytotoxic studies of the synthesized copolymers have been carried out (Table 4). It has been found that CL-PEG and *rac*-LA-PEG copolymeric matrices are not toxic to all test bionts, because the sample is considered to be toxic when the percent of the toxicity effect (PE) is greater than 20.

In the further step, hydrogels were obtained from the synthesized CL-PEG and *rac*‑LA-PEG copolymers, which were cross-linked with HDI. Sn(Oct)_2_ was used as polyaddition catalysts. The crosslinking reaction parameters are shown in Table 5.

Finally, hydrogel DDSs of 5-FU have been obtained by the incorporation method. The mean weight of the developed devices was about 500 mg, corresponding to approximately 20 mg of 5-FU (5-FU content was adjusted to about 5%).

**Figure 2:**
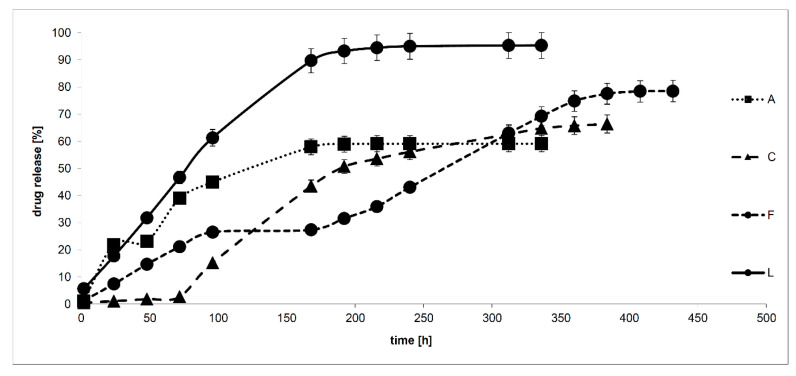
5‑fluorouracil (5-FU) release profile from the CL-PEG-HDI and *rac*-LA-PEG-HDI hydrogels. HDI: hexamethylene diisocyanate.

The kinetic release of 5-FU from the synthesized hydrogel devices A, C (CL-PEG-HDI), and F, L (*rac*‑LA-PEG-HDI) was determined at pH 7.0 ± 0.05 and 37 °C for 600 h (Figure 2). The samples for drug release kinetics evaluation were selected based on the copolymer type and water-absorption capacity of the crosslinked matrices. The ordinate of the plot was calculated based on the cumulative amount of drug released.

CL-PEG-HDI matrices (A, C) showed prolonged drug release profiles, up to 336 and 384 h for A and C, respectively. After 2 h of the experiment, the 5-FU cumulative release was about 1%. Subsequently, for the A matrix, the release rate increased significantly up to 22% after 24 h, reaching plateau after 168 h (59% of 5‑FU was released at this time). In the case of the C matrix, the release rate remained constant up to 72 h and then increased rapidly to 51% after 192 h. After 334 h of the experiment, the cumulative 5-FU release was 66%.

Cumulative release plots for *rac*‑LA-PEG-HDI matrices (F, L) reveal more controlled and closer to zero-order kinetic drug release profiles. The release rate appears to have been maintained throughout the experiment and decreased slightly after 360 h for the F matrix. The cumulative drug release rate was 78% after 432 h of incubation. A similar release profile was observed for the L matrix, but in this case, the release rate was 61% after 96 h compared to 27% for the F matrix. The drug release rate was maintained up to 192 h when the cumulative release rate reached plateau after 192 h (93% of 5-FU was released at that time).

It was observed that an increase in HDI/polymer and PEG 600/polymer ratios generally increased the release rate for *rac*‑LA-PEG-HDI hydrogels. Higher PEG 600 content increases the hydrophilicity of the hydrogel, which increases the water absorption capacity, facilitates the penetration of water molecules into the inner layers of the hydrogel, and elevates the release rate of the hydrophilic 5-FU. Although, when the HDI/polymer and PEG 600/polymer ratios were lower, the drug release kinetics became closer to zero-order kinetics, as it was observed for F (zero-order kinetics) and L (first-order kinetics) hydrogels. It is presumed that in the studied case, the decrease in HDI and PEG 600 content increase the significance of erosive release mechanisms with a simultaneous decrease in the amount of diffusion mechanisms, which was confirmed by the *n* values from the Korsmeyer–Peppas model (0.741 for zero-order F hydrogel, compared to 0.601 first-order L hydrogel). For *rac*‑LA-PEG-HDI hydrogels, the significant effect of HDI/polymer and PEG 600/polymer ratios was not observed.

The 5-FU release data points were subjected to a zero-order, first-order and Korsmeyer–Peppas models to evaluate the kinetics and the drug release mechanism from the obtained hydrogel carriers.

The *R*^2^ values for the zero-order kinetics model were relatively low, namely 0.7407 for A and 0.8259 for L hydrogels. Similarly, the *R*^2^ value for the first-order kinetics model was too low and was not identified with the A matrix. Based on the data in Table 6, it can be concluded that the drug was released with first-order kinetics rather from C (*R*^2^ = 0.9575) and L (*R*^2^ = 0.9101) matrices. In turn, 5-FU was released with zero-order kinetics from the F matrix (*R*^2^ = 0.9712).

In the Korsmeyer–Peppas model, the value of n characterized the drug release mechanism. In the case of A, F, and L hydrogels, the *n* value corresponds to the non-Fickian transport (0.45 < n = 0.89). The values of R^2^ for A, F, and L matrices were above 0.9. As shown in Table 6, the *R*^2^ value for C hydrogel was very small, and the mechanism of drug release cannot be specified.

Although hydrogels are typically characterized by relatively rapid drug release with not zero-order kinetics, as established in our work, systems appear to have slightly different properties due to extended polyester chain fragments, which are relatively hydrophobic. In addition, the developed hydrogels were chemically crosslinked with HDI, which further enhanced the hydrophobicity of the system. As a result, the overall hydrophobicity of the matrices was substantial, and the water permeability has been reduced. For this reason, the amount of drug released was relatively low, and release kinetics were more controlled and closer to zero order. Furthermore, according to the Korsmeyer–Peppas mathematical models, the drug release mechanism was defined as non‑Fickian diffusion (in most cases), involving diffusion and erosion processes.

## 4. Conclusions

New hydrogels as 5-FU carriers have been synthesized and characterized. The hydrogels were obtained from CL-PEG or *rac*‑LA-PEG copolymers and HDI as a crosslinking agent. The matrices have been successfully synthesized through the ROP of CL and *rac*‑LA in the presence of Zr(Oct)_4_ as catalyst. The Zr-based catalyst was active when the reactions were carried out both in solvent and “in bulk”. Due to the significantly lower yield of the products obtained in the solvent process, the method “in bulk” was established as the preferred method. The structure of the copolymers has been confirmed by NMR spectroscopy. Intensive intermolecular transesterification processes have been observed during *rac*‑LA polymerization, making it impossible to clearly establish the stereoselectivity of Zr(Oct)_4_ in the ROP process. The microstructure of the *rac*‑LA-PEG copolymers obtained was described as atactic. *T* values were in the range of 0.32–0.72 for *rac*‑LA-PEG copolymers obtained by the bulk method and tend to be independent of the time and temperature process (up to 140 °C). The optimal reaction conditions for CL-PEG and *rac*‑LA-PEG copolymers synthesis were defined as 140 °C and 24 h, and bulk polymerization was the preferred process.

It was observed that an increase in HDI/polymer and PEG 600/polymer ratios generally increases the 5‑FU release rate. Although, when the above mentioned ratios are lower, the drug release kinetics become closer to zero-order kinetics.

5-FU was released from the crosslinked CL‑PEG and *rac*‑LA-PEG hydrogels in a controlled and prolonged manner (up to 432 h). The release kinetics was defined as first or zero order. The release rate was dependent on the type of copolymer and the degree of crosslinking. In general, higher matrix hydrophilicity increases the release rate of the hydrophilic drug from the obtained hydrogel matrices, which is correlated with higher water absorption ability and improved access of the water molecules to the interior layers of the matrix. The release mechanism was defined as non‑Fickian transport.

In summary, the mentioned synthesis appears to be suitable for the preparation of hydrogels for biomedical applications; the synthesized products were non-toxic. Moreover, according to the literature, the system is considered to be biodegradable [33,34]. The release of 5-FU from hydrogel matrices has been prolonged and controlled, making the developed materials promising drug delivery systems for antitumor therapy.

## Figures and Tables

**Table 1:** Copolymerization of CL and PEG in the presence of Zr(Oct)_4._

Sample	Method	Temp. (°C)	Time (h)	% mol ^a^ CL	% mol ^a^ PEG	*M_n_*^b^ (g/mol)	*Đ* ^c^
**A-MS-1**	toluene	80	44	78	22	7100	1.68
**A-MS-2**	toluene	80	44	81	19	6900	1.72
**A-MS-3**	toluene	80	24	71	29	6600	1.64
**A-MS-4**	toluene	60	44	68	32	3700	1.61
**A-MS-5**	toluene	60	24	75	25	2800	1.49
**A-MS-11**	bulk	100	45	93	7	9600	2.03
**A-MS-12**	bulk	100	24	90	10	8400	1.89
**A-MS-13**	bulk	100	27	92	8	8600	1.86
**A-MS-14**	bulk	140	24	94	6	9500	1.94
**A-MS-15**	bulk	140	48	95	5	10,600	2.11
**A-MS-21**	bulk	160	24	78	22	10,400	2.06
**A-MS-22**	bulk	160	42	81	19	12,300	2.26
**A-MS-23**	bulk	160	48	81	19	13,600	2.42
**A-MS-24**	bulk	120	24	84	16	8300	1.71
**A-MS-25**	bulk	120	48	81	19	8600	1.76
**A-MS-31**	bulk	140	1	92	8	1800	1.34
**A-MS-32**	bulk	140	2	96	4	2100	1.38
**A-MS-33**	bulk	140	3	97	3	2600	1.45
**A-MS-34**	bulk	140	4	94	6	4200	1.54

^a^—percent molar content of mer in the polymer chain (determined by ^1^H NMR); ^b^—average molar mass determined by gel permeation chromatography (GPC); ^c^—dispersity determined by GPC.

**Table 2:** Copolymerization of *rac*-lactide (*rac*-LA) and PEG in the presence of Zr(Oct)_4_.

Sample	Method	Temp. (°C)	Time (h)	% mol ^a^ CL	% mol ^a^ PEG	*M_n_*^b^ (g/mol)	*Đ* ^c^	*T*^d^ (%)
**A-MS-6**	toluene	80	44	64	36	3700	1.76	72
**A-MS-7**	toluene	80	44	64	36	3300	1.69	72
**A-MS-8**	toluene	80	24	55	45	2300	1.89	≈100
**A-MS-9**	toluene	60	44	60	40			≈100
**A-MS-10**	toluene	60	24	62	38			≈100
**A-MS-16**	bulk	140	48	95	5	8900	2.26	32
**A-MS-17**	bulk	140	24	95	5	7200	1.96	40
**A-MS-18**	bulk	140	27	95	5	7500	2.41	40
**A-MS-19**	bulk	140	45	95	5	8200	2.29	48
**A-MS-20**	bulk	140	48	84	16	9700	2.31	48
**A-MS-26**	bulk	160	24	85	15	9400	2.16	64
**A-MS-27**	bulk	160	42	84	16	10,300	2.56	72
**A-MS-28**	bulk	160	48	84	16	11,700	2.64	72
**A-MS-29**	bulk	120	24	85	15	5400	1.62	48
**A-MS-35**	bulk	160	1	84	16	1100	1.29	0
**A-MS-36**	bulk	160	2	98	2	1600	1.48	48
**A-MS-37**	bulk	160	3	96	4	2400	1.54	48
**A-MS-38**	bulk	160	4	97	3	2800	1.59	56

^a^—percent molar content of mer in the chain (determined by ^1^H NMR); ^b^—average molar mass determined by GPC; ^c^—dispersity determined by GPC; ^d^—transesterification coefficient determined by ^13^C NMR.

**Table 3:** Synthesis of ε‑caprolactone‑poly(ethylene glycol) (CL-PEG) and rac-LA-PEG copolymers in a larger scale.

Sample	Copolymer	Temp. (°C)	Time (h)	% mol ^a^ CL	% mol ^a^ PEG	*M_n_*^b^ (g/mol)	*Đ* ^c^	*T*^d^ (%)
A‑MS‑39	CL‑PEG	140	24	93	7	6 800	1.73	
A‑MS‑40	*rac*‑LA‑PEG	140	24	96	4	10 800	1.98	32

^a^—percent molar content of mer in the chain (determined by ^1^H NMR); ^b^—average molar mass determined by GPC; ^c^—dispersity determined by GPC; ^d^—transesterification coefficient determined by ^13^C NMR.

**Table 4:** Cytotoxicity of the obtained copolymers.

Sample	Copolymer	Microtox^®^		Spirotox^®^		
		PE ^a^ (%)		Test Reactions		
		15 min	30 min	1 h	24 h	48 h
A‑MS‑39	CL‑PEG	13 ± 1	18 ± 1	none	none	none
A‑MS‑40	*rac‑*LA‑PEG	8 ± 1	6 ± 1	none	none	none

^a^—percent change in luminescence compared to the control.

**Table 5:** Crosslinking reaction parameters.

Copolymer	Sample	PEG 600/Copolymer (mol/mol)	HDI/Copolymer (mol/mol)	Copolymer Weight (g)	Water Absorption Capacity (%)
CL‑PEG	A	1	4.1	0.5325	57
	B	1	4.1	0.5214	21
	C	2	4.1	0.5729	16
	G	3	4.1	0.685	15
	H	7	8.3	0.898	14
	I	5	8.3	0.958	9
*rac*‑LA‑PEG	D	1	6.6	0.2649	114
	E	0.5	6.6	0.2829	84
	F	2	6.6	0.3597	107
	J	3	6.6	0.2275	291
	K	7	13.2	0.2143	397
	L	9	13.2	0.2022	470

**Table 6:** Analysis data of 5-FU release from hydrogels.

No.	Zero-Order Model	First-Order Model	Korsmeyer–Peppas Model		Drug Transport Mechanism
	*R* ^2^	*R* ^2^	*R* ^2^	*n*	
A	0.7407	0.7943	0.9178	0.752	non-Fickian transport
C	0.9157	0.9575	0.7786	1.037	unknown
F	0.9712	0.9325	0.9825	0.741	non-Fickian transport
L	0.8259	0.9101	0.9702	0.601	non-Fickian transport

## Data Availability

The data presented in this study are available on request from the corresponding author.

## Supplementary Materials

The following are available online at https://www.mdpi.com/1996-1944/14/1/98/s1, Table S1: HPLC gradient for 5-FU analysis; Figure S1: The ^1^H NMR spectrum of CL-PEG (in CDCl_3_); Figure S2: The ^13^C NMR spectrum of CL-PEG (in CDCl_3_); Figure S3: The ^1^H NMR spectrum of *rac*-LA-PEG (in CDCl_3_); Figure S4: The ^13^C NMR spectrum of *rac*-LA-PEG (in CDCl_3_).
