# Supplementary material for: Hydrogels Based on Poly(Ether-Ester)s as Highly Controlled 5-Fluorouracil Delivery Systems—Synthesis and Characterization
Source: Materials (Basel). 2020 Dec 28;14(1):98. doi: 10.3390/ma14010098 (PMC7795999; doi:10.3390/ma14010098)
Supplement: Supplementary file 1 [file materials-14-00098-s001.pdf]

materials

Supplementary Materials

Hydrogels Based on Poly(Ether-Ester)s as Highly Controlled 5-Fluorouracil Delivery Systems—Synthesis and Characterization

Adam Kasiński [1], Monika Zielińska-Pisklak [1], Ewa Oledzka [1], Grzegorz Nałęcz-Jawecki [2], Agata Drobniewska [2], Marcin Sobczak [1,]*

[1] Department of Biomaterials Chemistry, Chair of Analytical Chemistry and Biomaterials, Faculty of Pharmacy, Medical University of Warsaw, 1 Banacha St., 02-097 Warsaw, Poland; adam.kasinski@wum.edu.pl (A.K.); monikapisklak@o2.pl (M.Z.-P.); ewa.oledzka@wum.edu.pl (E.O.)
[2] Department of Environmental Health Sciences, Faculty of Pharmacy, Medical University of Warsaw, 1 Banacha St., 02-097 Warsaw, Poland; grzegorz.nalecz-jawecki@wum.edu.pl (G.N.-J.); agata.drobniewska@wum.edu.pl (A.D.)
* Correspondence: marcin.sobczak@wp.pl or marcin.sobczak@wum.edu.pl

Citation: Kasiński, A.; Zielińska-Pisklak, M.; Oledzka, E.; Nałęcz-Jawecki, G.; Drobniewska, A.; Sobczak, M. Hydrogels based on poly(ether-ester)s as highly controlled 5-fluorouracil delivery systems—synthesis and characterization. *Materials* **2020**, *14*, 98. https://doi.org/10.3390/ma14010098

Received: 11 November 2020
Accepted: 23 December 2020
Published: 28 December 2020

Publisher's Note: MDPI stays neutral with regard to jurisdictional claims in published maps and institutional affiliations.

Copyright: © 2020 by the authors. Licensee MDPI, Basel, Switzerland. This article is an open access article distributed under the terms and conditions of the Creative Commons Attribution (CC BY) license (http://creativecommons.org/licenses/by/4.0/).

Table S1. HPLC gradient for 5-FU analysis.

Time (min)	Phase A (%) (H_2O + 0.1 % TFA)	Phase B (%) (ACN + 0.1 % TFA)
**0**	99	1
**7**	96	4
**12**	20	80
**15**	20	80
**20**	99	1
**25**	99	1

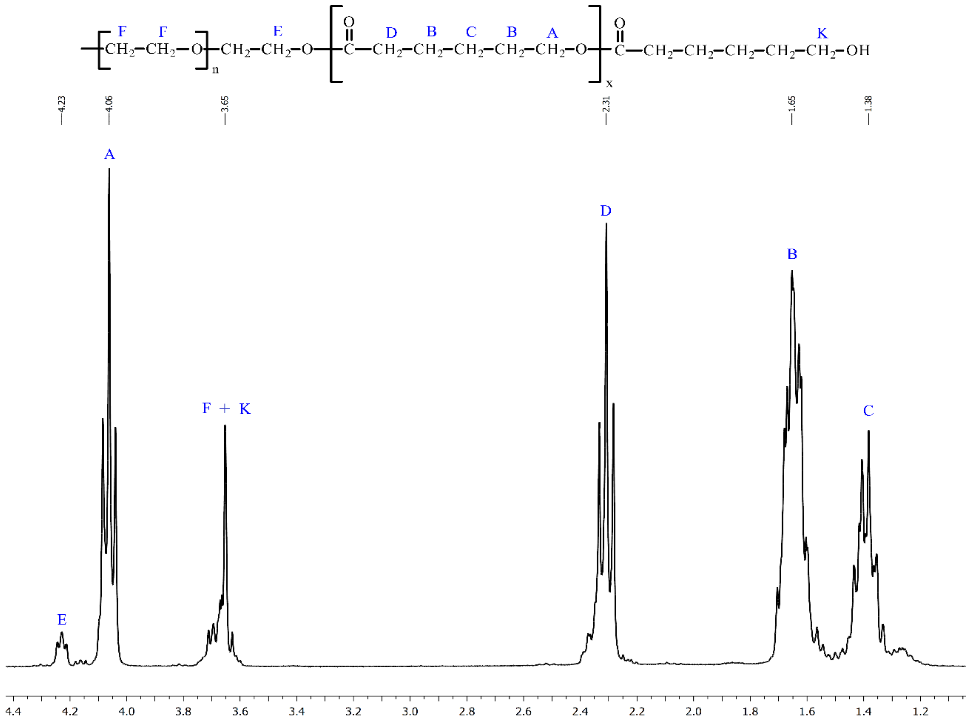

Figure S1. The ^1H NMR spectrum of CL-PEG (in $CDCl_3$).

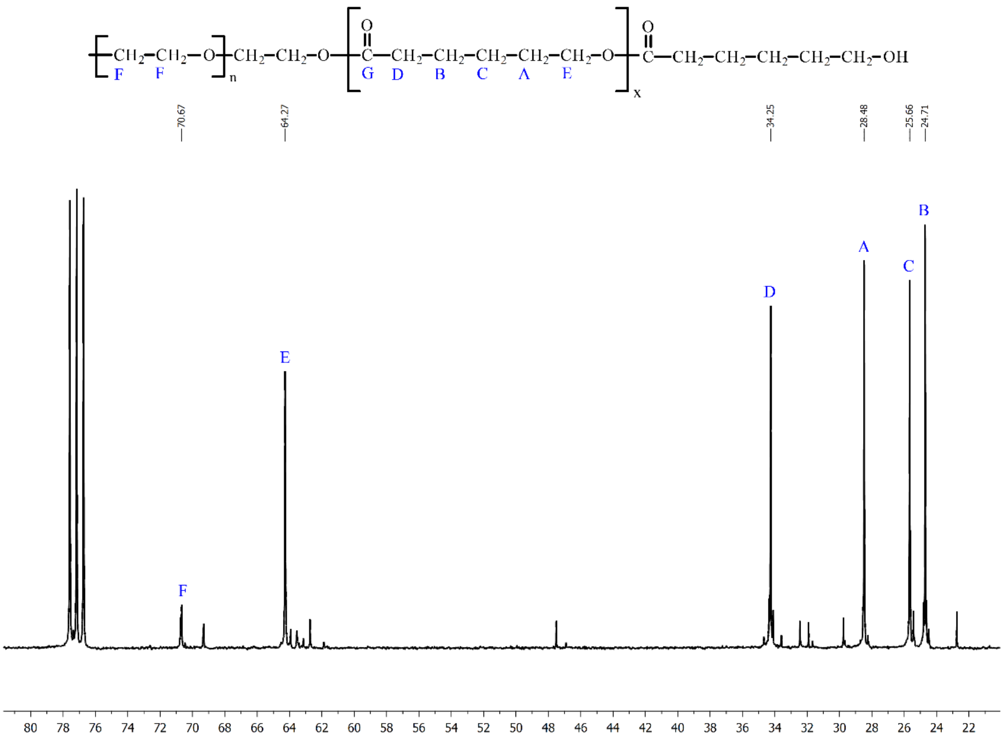

Figure S2. The ^{13}C NMR spectrum of CL-PEG (in $CDCl_3$).

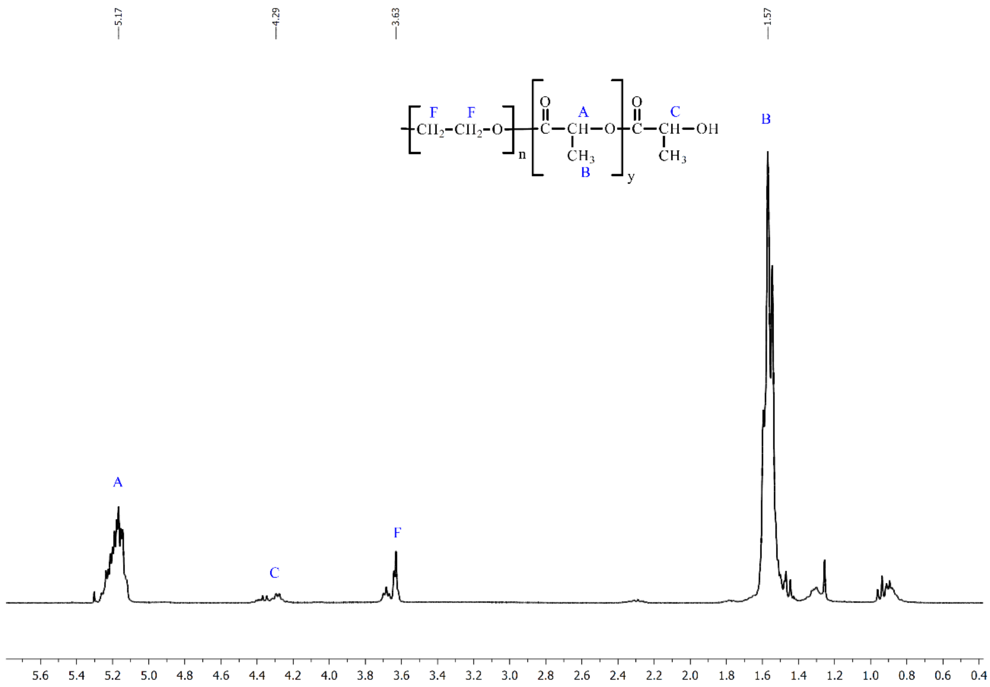

Figure S3. The ^{1}H NMR spectrum of *rac*-LA-PEG (in $CDCl_3$).

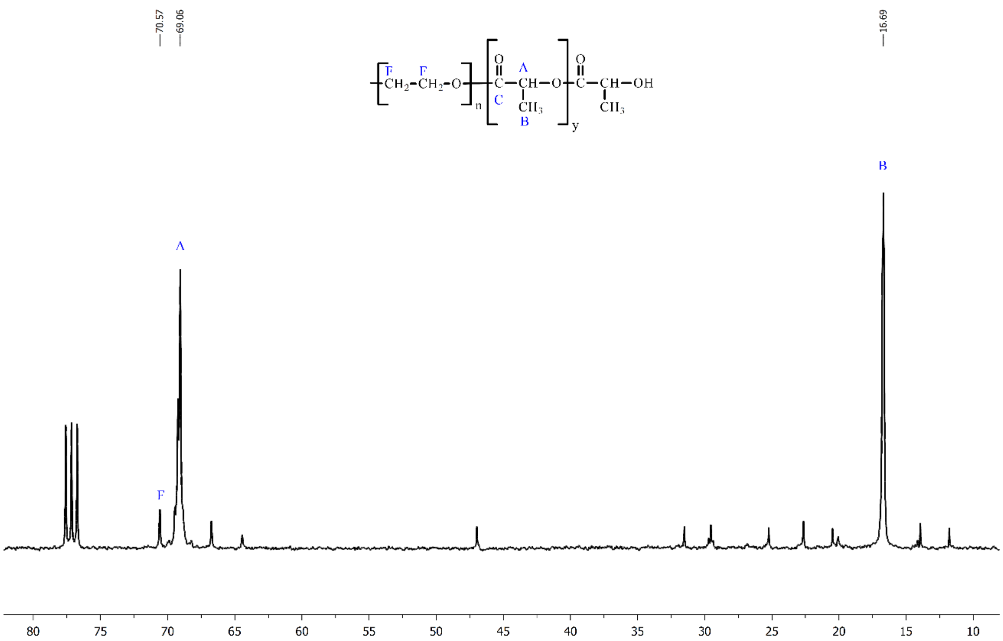

Figure S4. The ^{13}C NMR spectrum of *rac*-LA-PEG (in $CDCl_3$).
